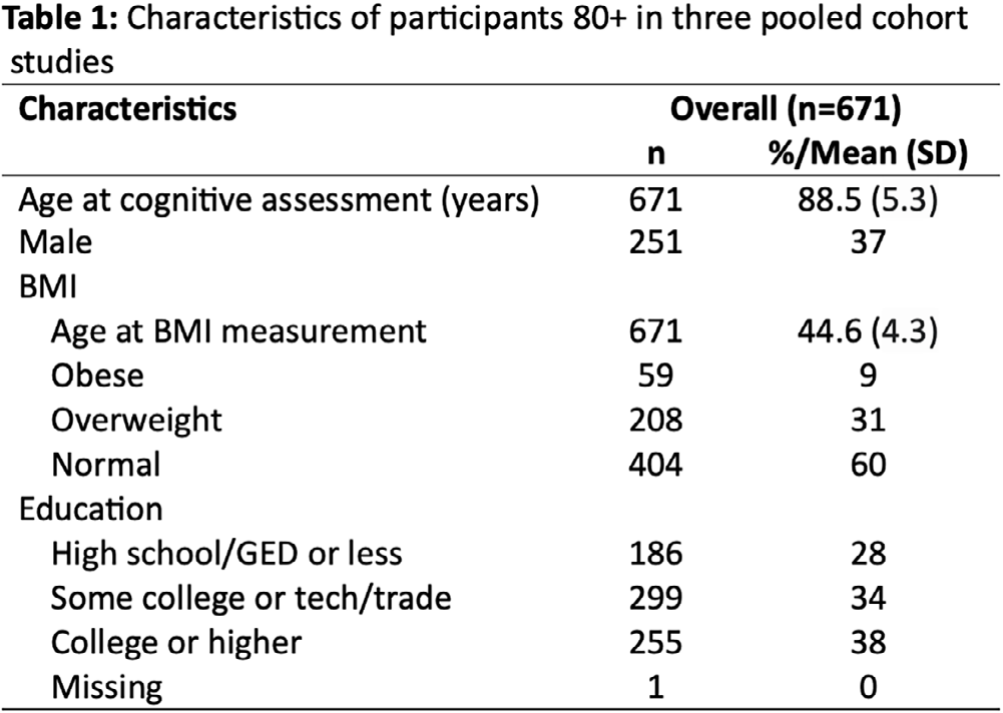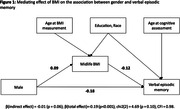# The mediating effect of BMI on the association between gender and domain specific cognition in a diverse cohort of individuals 80+

**DOI:** 10.1002/alz.092621

**Published:** 2025-01-09

**Authors:** Claire C. Meunier, Joseph Roscoe, Paola Gilsanz, María M. M. Corrada, Kristen M. George, M. Maria Glymour, Elizabeth Rose Mayeda, Brandon E Gavett, Alexander Ivan B. Posis, Rachel A. Whitmer

**Affiliations:** ^1^ University of California, Davis, Davis, CA USA; ^2^ Kaiser Permanente Northern California Division of Research, Oakland, CA USA; ^3^ University of California, Irvine, Irvine, CA USA; ^4^ Boston University School of Public Health, Boston, MA USA; ^5^ UCLA Fielding School of Public Health, University of California, Los Angeles, CA USA; ^6^ University of California Davis, Sacramento, CA USA

## Abstract

**Background:**

Gender differences are often associated with specific cognitive domains. Additionally, gender may be associated with BMI through biological and social differences. We evaluated if mid‐life BMI mediated the effect of gender on domain specific cognition in an ethnoracially diverse population of individuals 80+.

**Method:**

Data from three ongoing cohort studies, LifeAfter90, Kaiser Healthy Aging and Diverse Life Experiences, and Study of Healthy Aging in African Americans, was pooled to include participants aged 80+ (n = 671). Baseline verbal episodic memory (VEM), executive function (EF), and semantic memory (SM) were measured using the z‐standardized Spanish English Neuropsychological Assessment Scales. Mid‐life BMI was categorized as normal (18.5‐24.9 kg/m^2^), overweight (25‐29.9 kg/m^2^), and obese (30 kg/m^2^) using the first clinical measurement at ages 40‐60. Structural equation models were used to estimate the indirect effect (IDE) of mid‐life BMI category on the association between gender and cognition adjusting for age at baseline cognitive assessment, age at BMI measurement, racial/ethnic identity, and education.

**Result:**

Participants were 37% male, 17% Asian, 35% Black, 12% Latino, 33% White, 3% other racial/ethnic group with a mean age of 88.5±5.3 at baseline cognitive assessment and a mean age at mid‐life BMI measure of 44.6±4.3 years (table 1). Midlife obesity (9%) and overweight (31%) were common. The total effect of gender (ref = women) on each cognitive domain was significant (VEM: ‐0.19, p < 0.001; EF: ‐0.12, p = 0.001; SM: 0.202, p < 0.001). Five percent (p‐value = 0.07) of the association between gender and VEM was explained by BMI category (IDE (β = ‐0.01; p‐value = 0.06) divided by total effect (β = ‐0.19; (p‐value<0.001); Figure 1). BMI did not mediate the association between gender and SM (β(IDE) = ‐0.003;p‐value = 0.29) or between gender and EF (β(IDE) = ‐0.005; p‐value = 0.19).

**Conclusion:**

In this ethnoracially diverse population of individuals aged 80+, mid‐life BMI marginally mediated the association between gender and baseline VEM, while it did not mediate the associations with EF and SM. Additionally, men had lower EF and VEM but higher SM compared to women. The effect of mid‐life BMI on the association between gender and cognition may vary by cognitive domain, suggesting that mid‐life BMI may impact specific late‐life cognitive functions that are associated with VEM.